# Protein Palmitoylation in Leukocyte Signaling and Function

**DOI:** 10.3389/fcell.2020.600368

**Published:** 2020-10-28

**Authors:** Xiaoyuan Yang, Victor Chatterjee, Yonggang Ma, Ethan Zheng, Sarah Y. Yuan

**Affiliations:** ^1^Department of Molecular Pharmacology and Physiology, Morsani College of Medicine, University of South Florida, Tampa, FL, United States; ^2^Department of Surgery, Morsani College of Medicine, University of South Florida, Tampa, FL, United States

**Keywords:** palmitoylation, protein function, leukocyte function/activation, signal transduction, DHHC palmitoyl transferases, leukocyte behaviors

## Abstract

Palmitoylation is a post-translational modification (PTM) based on thioester-linkage between palmitic acid and the cysteine residue of a protein. This covalent attachment of palmitate is reversibly and dynamically regulated by two opposing sets of enzymes: palmitoyl acyltransferases containing a zinc finger aspartate-histidine-histidine-cysteine motif (PAT-DHHCs) and thioesterases. The reversible nature of palmitoylation enables fine-tuned regulation of protein conformation, stability, and ability to interact with other proteins. More importantly, the proper function of many surface receptors and signaling proteins requires palmitoylation-meditated partitioning into lipid rafts. A growing number of leukocyte proteins have been reported to undergo palmitoylation, including cytokine/chemokine receptors, adhesion molecules, pattern recognition receptors, scavenger receptors, T cell co-receptors, transmembrane adaptor proteins, and signaling effectors including the Src family of protein kinases. This review provides the latest findings of palmitoylated proteins in leukocytes and focuses on the functional impact of palmitoylation in leukocyte function related to adhesion, transmigration, chemotaxis, phagocytosis, pathogen recognition, signaling activation, cytotoxicity, and cytokine production.

## Introduction

Leukocytes are critical components of innate and adaptive immunity by eradicating microbes and potentially harmful cells or substances. In addition to combating infection, leukocytes are also involved in the pathogenesis of many diseases, including cancer, neurological disorders, and cardiovascular diseases. In order to ensure effective leukocyte function without causing unwanted damages to normal tissues, the cascades of leukocyte responses are precisely controlled by many leukocyte proteins such as adhesion molecules, surface receptors, co-receptors, signaling effectors, adaptor proteins, cytokines, and chemokines (Yadav et al., 2003). Aberrant function of any of these proteins may lead to abnormal immune responses. Leukocyte proteins undergo a variety of post-translational modifications (PTMs) to ensure fine-tuned regulation and functional diversity. These modifications include acylation, phosphorylation, ubiquitination, glycosylation, nitrosylation, methylation, and proteolysis (Liu et al., 2016). Among these PTMs, protein palmitoylation is a prominent type of acylation that has gained increasing recognition for its roles in regulating leukocyte signaling and behaviors.

Palmitoylation belongs to a subtype of fatty acid modification called S-acylation, involving covalent attachment of 16-carbon palmitic acid to one or more cysteine residues of a protein through a thioester linkage (R-S-CO-R’). In 1979, palmitoylation was first identified by Schmidt and Schlesinger (1979). The source of palmitic acid is the cytosolic pool of palmitoyl-CoA (Resh, 2006a). The addition of palmitic acid is an enzyme-catalyzed reaction (Blaskovic et al., 2013). Palmitoylation differs from other types of lipid modifications (e.g., myristoylation and prenylation) in that palmitoylation is reversible and dynamically regulated. The attached palmitate is readily removed when the thioester linkage is cleaved. Cycles of palmitoylation and de-palmitoylation are precisely regulated in a fashion similar to phosphorylation and ubiquitination. The reversibility of palmitoylation allows for the dynamic regulation and fine-tuning of protein function and activities in leukocytes.

Although protein palmitoylation was discovered about 40 years ago, the field has substantially progressed only in recent years after the breakthrough of non-radioactive detection of palmitoylated proteins. Palmitoylation has since been extensively studied in cancer and neurological disorders (Kang et al., 2008; Ko and Dixon, 2018); however, research on palmitoylation regulation in leukocyte function is in its infancy, with many molecular mechanisms remaining largely unexplored. Recently, methodological advances have made it possible to perform sensitive and reliable large-scale palmitoyl proteome profiling in different subtypes of leukocytes, including monocyte/macrophages, dendritic cells, T cells and B cells, as well as in cells directly interacting with leukocytes, such as endothelial cells (ECs; Martin and Cravatt, 2009; Merrick et al., 2011; Ivaldi et al., 2012; Marin et al., 2012; Chesarino et al., 2014). These palmitoyl proteomic approaches have identified an array of palmitoylated proteins, including but not limited to cytokine/chemokine receptors CCR5, TNFR, and INFAR; adhesion molecules PECAM-1 and JAM-C; pattern recognition TLR/MYD88 complex; scavenger receptor CD36; T cell co-receptors CD4 and CD8; Src family kinases Lck and Fyn; transmembrane adaptor proteins linker of activated T cells (LAT) and PAG; signaling effectors Gα, Ras, and phospholipase C; and Ca^2+^ regulator IP3R. This growing list of palmitoylated proteins highlights the functional significance of palmitoylation in leukocytes. In this review, we discuss the latest advances in palmitoylation-mediated regulation of protein function and its impact on leukocyte signaling and behaviors.

## Palmitoylation in Protein Function and Signal Transduction

**FIGURE 1:**
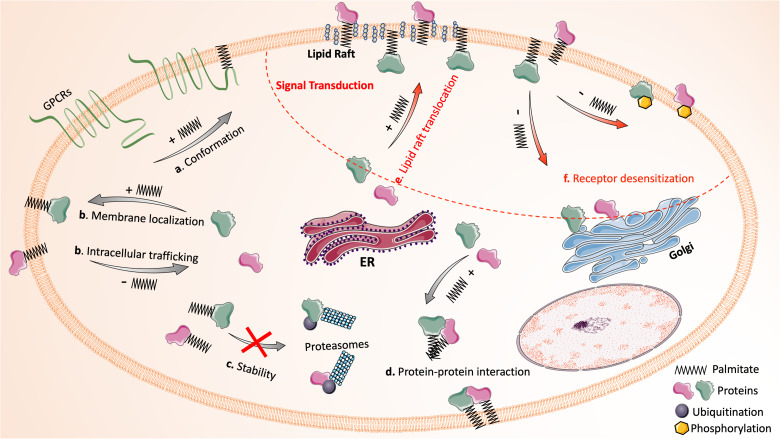
Protein palmitoylation dynamically regulates protein function and signal transduction. The reversible attachment of palmitic acid affects protein function in many ways: **(a)** palmitoylation alters the conformation or structure of a protein (e.g., the fourth intracellular loop of GPCRs that is formed by inserting palmitate into the plasma membrane); **(b)** palmitoylation mediates protein intracellular trafficking and ensures stable membrane localization of proteins; **(c)** palmitoylation stabilizes proteins by preventing misfolding and ubiquitination-mediated degradation; **(d)** palmitoylation facilitates protein-protein interaction directly and indirectly. In addition, the palmitoylation and de-palmitoylation cycle has critical impact on signal transduction (upper right region circled by red dash line): **(e)** many signaling proteins require palmitoylation for their translocation into lipid rafts. **(f)** De-palmitoylation mediates receptor desensitization following activation by promoting the phosphorylation of receptors or the translocation of membrane signaling proteins to cytoplasm. Images of proteins and organelles were obtained from Smart Servier Medical Art (https://smart.servier.com).

The regulatory mechanisms of palmitoylation in protein function and signal transduction are summarized in Figure 1.

### Subcellular Effects

Covalent attachment of palmitic acid to proteins results in increased protein hydrophobicity and, therefore, affects protein function mainly through four mechanisms: altering conformation/structure, directing intracellular trafficking or compartmentalization, regulating stability/half-life, and influencing protein-protein interactions. Firstly, palmitoylation plays a critical role in regulating protein conformation. Many G-protein coupled receptors (GPCRs) in leukocytes require palmitoylation for their proper conformation. The insertion of the attached palmitate into plasma membrane creates the fourth loop of GPCRs between the cytoplasmic end of the seventh α-helix and palmitate insertion, which is essential for the propagation of GPCR signals (Chini and Parenti, 2009). Secondly, palmitoylation regulates protein trafficking and localization, as typically seen with small GTPase Ras, one of the most studied palmitoylated substrates. The palmitoylation and de-palmitoylation cycle dynamically regulates Ras shuttling between Golgi and plasma membrane (Hornemann, 2015). Likewise, many transmembrane or membrane-associated proteins rely on palmitoylation for their stable membrane anchor (Greaves and Chamberlain, 2007). Thirdly, emerging evidence has shown that palmitoylation can stabilize proteins and increase their half-life. Mutation of the palmitoylation site greatly shortens the life span of many proteins, as such proteins undergo misfolding during protein synthesis or become more susceptible to ubiquitination-mediated degradation (Resh, 2006a). Finally, palmitoylation modulates protein-protein interactions both directly and indirectly. The long hydrocarbon chain of the attached palmitic acid can interact directly with that of another protein. Palmitoylation also promotes the clustering of proteins in specialized membrane microdomains, which selectively brings specific proteins into proximity and facilitates their interactions indirectly (Blaskovic et al., 2013).

### Impact in Signal Transduction

A growing list of receptors and signaling proteins in leukocytes have been recognized as palmitoylation substrates (Resh, 2006a). Their function can be modulated by palmitoylation in similar manners as stated above. Additionally, palmitoylation facilitates the sequestering of receptors, signaling proteins, and adaptor proteins into lipid rafts for effective signal transduction (Bijlmakers and Marsh, 2003). Lipid rafts are liquid-ordered microdomains in the external leaflet of plasma membrane enriched with cholesterol and glycosphingolipids; they are highly dynamic and serve as a platform for many signal transduction processes in leukocytes (Alonso and Millan, 2001). For example, the signaling proteins for T-cell activation, including the T cell receptor, its co-receptors (CD4 and CD8), Src-family kinases (Lyn and Fyn), and adaptor proteins are all clustered in lipid rafts of T cells (Kabouridis, 2006). As a saturated fatty acid, palmitate prefers to insert into liquid-ordered raft domains rather than the bulk plasma membrane (Resh, 2013). Palmitoylation-dependent segregation of proteins in lipid rafts is essential for the amplification of signal propagation in activated cells while preventing the signal activation in resting cells. Another unique function of palmitoylation in regulating signal transduction is mediating receptor desensitization and internalization (Resh, 2006a). Upon activation, many receptors become desensitized as a result of phosphorylation mediated by GPCR kinases (GRKs), the function of which is greatly enhanced by palmitoylation (Stoffel et al., 1998). Meanwhile, de-palmitoylation of receptors has been proved to increase the accessibility of their phosphorylation sites to kinases (Moffett et al., 1993; Qanbar and Bouvier, 2003). Thus, palmitoylation and de-palmitoylation precisely control signaling events in a spatial-temporal context, regulating many aspects of leukocyte function.

As the fundamental signaling events in many leukocytes, GPCR signal transduction is subjected to palmitoylation-dependent regulation at many levels. In addition to GPCRs themselves being affected by palmitoylation in the aforementioned manner, various GPCR downstream effectors are also substrates of palmitoylation. Upon GPCR activation, Gα subunit dissociates from Gβγ subunit, and their fragments subsequently activate three major signal transduction cascades: (1) *Adenylate cyclase cascade*, which catalyzes the conversion of ATP to cAMP and then activates PKA and its downstream effectors; (2) *Phospholipase c (PLC) cascade*, which promotes the conversion of phosphatidylinositol 4,5-bisphosphate [PI(4,5)P2] to diacylglycerol (DAG) and inositol 1,4,5-trisphospate (IP3). IP3 then binds to IP3 receptor (IP3R) localized on ER and induces the release of Ca^2+^ into cytoplasm. DAG and increased Ca^2+^ are both capable of activating PKC; (3) *Phosphatidylinositol 3 kinase (PI3K) cascade*, which hydrolyzes PI(4,5)P2 to produce phosphatidylinositol 3,4,5-triphosphate (PIP3), an effective activator of AKT (also known as PKB; Hilger et al., 2018; Lammermann and Kastenmuller, 2019; Wang X. et al., 2019). Many effector proteins of the GPCR signaling cascades, for example, Gα, PLC and IP3R, require palmitoylation for their proper localization and function. Gαq is palmitoylated at Cys9 and 10, while Gαs is palmitoylated at Cys3 (Wedegaertner et al., 1993). Gαq and Gαs with mutation at these cysteine sites exist in their soluble form in cytoplasm rather than their primary location in lipid rafts. Palmitoylation-defective Gαq and Gαs are incapable of coupling with GPCRs and fail to induce the activation of PLC or adenylate cyclase, respectively (Wedegaertner et al., 1993). A separate study shows that the palmitate turnover rate of Gα increases upon Gα activation and dissociation from Gβγ; de-palmitoylation mediates the internalization of Gα to cytoplasm, where it is repalmitoylated (Ross, 1995). This activation-dependent de-palmitoylation of Gα is essential to the desensitization process following GPCR activation (Vogler et al., 2008).

Ras, which belongs to the small GTPases, also functions as a signaling effector. Ras is one of the earliest identified palmitoylation substrates (Magee et al., 1987). All three Ras isoforms (H-, N-, and K-Ras) undergo farnesylation at the C-terminal CAAX motif, allowing Ras to loosely insert into endomembrane structures. N-Ras and H-Ras, but not K-Ras, are further modified by attaching palmitates at one or two cysteines (Cys181 in N−Ras, Cys181 and 184 in H−Ras) adjacent to CAAX motif in Golgi (Hancock et al., 1990; Swarthout et al., 2005; Baumgart et al., 2010). The attached palmitate promotes the translocation of Ras to plasma membrane and facilities the stable membrane anchor of Ras. Reports show that palmitoylation-defective H-Ras is trapped in Golgi and unable to traffic to plasma membrane. Palmitoylation also influences the GTP-binding ability of Ras. Functional assay revealed that only palmitoylated N-Ras turns into the GTP-bound activated form in response to agonists (Song et al., 2013). The palmitate turnover rate of GTP-bound activated Ras is 25-fold higher than that of GDP-bound deactivated Ras (Baker et al., 2003). After de-palmitoylation, Ras traffics to Golgi, where it is palmitoylated again and redirected to plasma membrane for the next cycle.

## Palmitoylation Enzymes

The turnover of protein palmitoylation is generally much faster than the turnover of the particular protein being palmitoylated, indicating that the attachment of palmitate is dynamically regulated (Won et al., 2018). Protein palmitoylation is controlled by two opposing types of enzymes. Palmitoyl acyltransferases (PATs) catalyze the addition of palmitic acid, while the removal of palmitic acid is mediated by thioesterases, including palmitoyl protein thioesterases (PPTs), acyl-protein thioesterases (APTs) and α/β hydrolase domain-containing 17 proteins (ABHD17s; Won et al., 2018). Under the tight control of these enzymes, the palmitoylation and de-palmitoylation cycle normally occurs multiple times throughout the lifetime of a palmitoylated protein.

### Palmitoyl Acyltransferases

Palmitoyl acyltransferases contain a highly conserved Asp-His-His-Cys (DHHC) tetrapeptide motif in its cysteine-rich domain; thus these enzymes are also known as DHHCs or PAT-DHHCs (Tabaczar et al., 2017). DHHCs were first identified by Deschenes and Davis labs in 2002 (Lobo et al., 2002; Roth et al., 2002). So far, 23 DHHCs have been identified in humans (Rana et al., 2019). DHHCs are transmembrane proteins that span the membrane four to six times and locate on the cytosolic face of the membrane (Blaskovic et al., 2013). The conserved cysteine-rich domain serves as the catalytic center for the enzyme, directly involved in the palmitoyl transfer reaction (Tabaczar et al., 2017; Rana et al., 2019). The majority of DHHCs are localized in endoplasmic reticulum (ER; DHHC1, 6, 14, 19, 23, 24) or Golgi apparatus (DHHC 3, 4, 7, 8, 15, 17, 18) or both (e.g., DHHC 2, 9, 12, 13, 22), while several others (e.g., DHHC 5, 20, 21) can be found on plasma membrane (Korycka et al., 2012; Hornemann, 2015; Ko and Dixon, 2018). DHHCs catalyze palmitoylation in a two-step manner: (1) auto-palmitoylation reaction where the cysteine residue in DHHC motif binds with palmitoyl-CoA; (2) acyl transfer reaction where the palmitoyl group is transferred from DHHCs to cysteine residues of a protein substrate (Tabaczar et al., 2017). To date, the substrate specificity of DHHCs is poorly understood. Although the palmitoylated cysteines share some common characteristics, there is no consensus palmitoylation sequence (Salaun et al., 2010). It is speculated that the protein-protein interaction domain of DHHCs (e.g., ankyrin repeats in DHHC13/17, Src-homology 3 domain in DHHC6) plays critical roles in recognizing specific substrates (Fredericks et al., 2014; Verardi et al., 2017; Matt et al., 2019).

Accumulating studies have revealed the functional impact of DHHCs in leukocytes. For example, mice with DHHC21 deficiency display a reduced number of slow-rolling and adherent leukocytes in post-capillary venules during systemic inflammation (Beard et al., 2016). There is evidence for DHHC6- catalyzed MyD88 palmitoylation in neutrophils, which is necessary for TLR-NF-κB signaling (Kim et al., 2019). In dendritic cells, surface expression and proper function of TLR2 depend on the palmitoylation of TLR2 by DHHC 2, 3, 6, 7, or 15 (Chesarino et al., 2014). DHHC7 is responsible for the palmitoylation of Fas, thus regulating T cell apoptosis and differentiation (Rossin et al., 2015). Although beyond the current focus, it is worth pointing out that palmitoylation can affect the infectivity of various viruses and bacteria, including coronavirus, HIV, influenza, and B anthrax (Sobocinska et al., 2017). It has been reported that DHHC-mediated palmitoylation of the SARS-CoV spike protein facilitates the virus entry into host cells (Petit et al., 2007). This may also hold true for the SARS-Cov2 virus, as a study has reported the interaction of SARS-Cov2 with DHHC5 (Gordon et al., 2020).

### De-Palmitoylation Enzymes

Many signaling proteins in leukocytes, such as Ras and certain G protein subunits, undergo rapid de-palmitoylation upon activation, suggesting that de-palmitoylation enzymes participate in leukocyte responses (Won et al., 2018). So far, three classes of de-palmitoylation enzymes have been identified: PPTs, APTs and ABHD17s. ***PPTs*** (PPT1 and PPT2) mainly reside in lysosomes and mediate the removal of palmitate during the process of protein degradation (Resh, 2006a; Koster and Yoshii, 2019). PPT1 was the first identified enzyme that removes palmitate from a modified protein (Camp and Hofmann, 1993). Although PPT1 has been reported to show activities outside of lysosomes in certain neurons, it is unlikely that PPT1 contributes to the de-palmitoylation of cytoplasmic or plasma membrane proteins (Kim et al., 2008; Hornemann, 2015). PPT2 shares 18% of its amino acid sequence with PPT1 and shows a certain degree of cross-reactivity with PPT1 (Gupta et al., 2001; Kim et al., 2008; Cho and Park, 2016). However, PPT2 does not catalyze the de-palmitoylation of H-Ras as PPT1 does (Soyombo and Hofmann, 1997), suggesting a distinctive substrate specificity for PPT2. ***APTs*** (APT1 and APT2) are ubiquitously expressed serine hydrolases, localized predominantly in cytoplasm (Kong et al., 2013). APT1 (or LYPLA1) was first identified as a de-palmitoylation enzyme in 1998, based on its ability to remove [^3^H] palmitate from palmitoylated Gα and H-Ras (Duncan and Gilman, 1998). Further studies show that APT1 catalyzes de-palmitoylation of numerous leukocyte proteins, including linker of activated T cells (LAT), endothelial nitric oxide synthase (eNOS), regulator of G protein signaling protein (RGS), and synaptosome associated protein 23 (SNAP-23; Ladygina et al., 2011). APT2 exhibits 68% of amino acid sequence similarity to APT1 and has an almost identical 3D-structure to APT1 (Gadalla and Veit, 2020). APT2 seems to possess distinct substrate preference compared to APT1. Nevertheless, APT2 and APT1 are functionally redundant in de-palmitoylating certain proteins (e.g., Ras; Rusch et al., 2011). So far, little is known about the mechanism of APT substrate specificity. Several studies have investigated the regulatory mechanism and functional impact of APTs in leukocytes. The expression of APT1 in RAW264.7 macrophage-like cells decreases in response to LPS, while the expression of APT2 remains unaffected (Satou et al., 2010). A recent study of B cells shows that APTs are inhibited by miR-138 and -424, which exerts critical impacts on B cell apoptosis in a CD95-dependent mechanism (Berg et al., 2015). ***ABHD17s*** (ABHD17A, ABHD17B and ABHD17C) are mainly localized to plasma membrane of various cell types (Lin and Conibear, 2015; Won et al., 2018). The ABHD17s were first identified as the de-palmitoylase for N-Ras (Lin and Conibear, 2015). ABHD17s are palmitoylated at the N-terminal cysteine cluster, which is required for its plasma membrane localization and substrate interaction (Lin and Conibear, 2015). So far, there has been limited research to characterize the functional impacts and regulatory mechanisms of ABHD17s.

## Methods of Detecting Palmitoylation

Detection of palmitoylated protein has been technically challenging due to its reliance on time-consuming radioactive labeling. Recently, several methodological breakthroughs have been achieved for quantifying and imaging palmitoylated proteins, which greatly accelerate the research progress in this field. Currently, there are three major types of detection methods: radioactive metabolic incorporation, hydroxylamine-based acyl-exchange assay, and click chemistry-based metabolic labeling. All can be applied to detect palmitoylated proteins in leukocytes.

### Radioactive Metabolic Incorporation

Palmitoylated proteins can be metabolically labeled with palmitic acid incorporated with a radioisotope. Tritiated palmitate (or [^3^H] palmitate) is the most commonly used radioactive palmitate as it is commercially available and confirmed as reliable by many labs. This method consists of four steps: [^3^H] palmitate labeling, immunoprecipitation proteins, SDS-PAGE, and photofluorographic exposure (Resh, 2006b). When coupling it with a pulse-chase approach, [^3^H] palmitate labeling can be utilized to study the palmitate turnover of a specific protein substrate. For more than two decades, radioactive labeling has remained the gold standard for detecting palmitoylation (Yount et al., 2013). However, β particles emitted by [^3^H] are weak, thus it often requires several weeks to develop a clear X-ray image through photofluorography. The time-consuming and cumbersome nature of this method limits its use in many research labs, not to mention the hazards associated with handling radioactive materials. Later, a more energetic radioactive palmitate analog [^125^I]iodohexadecanoic acid (^125^I-IC16) was adapted to this method. [^125^I] generates gamma rays that are more penetrating, thus greatly shortening the exposure time to 24 h (Tsai et al., 2014). Other advances in radioactive labeling involve improving the sensitivity of X-ray films, which enables visualization of [^3^H] palmitate-labeled proteins in 1–3 days (Tsai et al., 2014).

### Hydroxylamine-Based Acyl-Exchange Assay

Hydroxylamine-based acyl-exchange assay is one of the two non-radioactive techniques invented to circumvent the drawbacks of radioactive labeling (Drisdel and Green, 2004). This method is comprised of four biochemical steps: (1) blocking the free thiols of extracted proteins; (2) cleaving the thioester linkage with hydroxylamine to liberate the previously palmitoylated cysteinyl thiols; (3) capturing the newly freed thiol groups with pyridyldithiol-biotin; (4) purifying the biotin-labeled proteins using streptavidin agarose beads (Wan et al., 2007; Brigidi and Bamji, 2013). More recently, acyl-biotin exchange assay (ABE) was modified to a simplified version called resin-assisted capture (RAC) in which proteins with hydroxylamine-liberated thiol groups are directly captured by pyridyldithiol-resin (Forrester et al., 2011). The acyl-exchange assays are more powerful when coupled with mass spectrometry-based proteomic studies. Unlike radioactive labeling that analyzes a single protein, acyl-exchange assays enable large-scale profiling of the proteome, leading to the discovery of numerous novel palmitoylation substrates in leukocytes (Ivaldi et al., 2012). Moreover, acyl-exchange assays are unique among all the palmitoylation detecting methods for their capability in providing a snapshot of the palmitoylated protein profile in cells. The major disadvantage of acyl-exchange assays lies within the fact that they are not compatible with pulse-chase experiments, as the entire acyl-exchange procedure is performed in protein lysates (Tsai et al., 2014).

### “Click Chemistry”-Based Metabolic Labeling

“Click chemistry”-based metabolic labeling is the second type of non-radioactive techniques used to detect palmitoylation. The principle behind this method is the “click chemistry” reaction, also known as Cu^1+^-catalyzed Huisgen cycloaddition reaction between azido- and alkynyl-groups (Hang et al., 2007; Charron et al., 2009; Hannoush and Arenas-Ramirez, 2009). The most commonly used alkynyl-palmitate analogs are ω-alkynyl-C16 (Alk-C16 or 15-hexadecynoic acid) and 17-octadecynoic acid (17-ODYA). “Click chemistry”-based metabolic labeling is most versatile in application compared to other methods due to the various types of azido-containing markers. First and foremost, palmitoylated proteins labeled with alkynyl groups can react with azido-fluorescent dye (e.g., Oregon Green 488 azide), which enables the robust imaging of global protein palmitoylation in cells (Hannoush and Arenas-Ramirez, 2009; Beard et al., 2016). Later, this imaging strategy was further improved by coupling with proximity ligation assay, which makes it possible to visualize a single palmitoylated protein and study its palmitoylation level and subcellular localization (Gao and Hannoush, 2014). Secondly, alkynyl-palmitoylated proteins can also react with azido-biotin for streptavidin enrichment, followed by either single protein analysis or large-scale LC-MS/MS proteomic analysis in leukocytes (Ladygina et al., 2011). Thirdly, alkynyl-palmitoylated proteins can bind to azido-IRDye 800, omitting the streptavidin enrichment step and allowing the direct detection of leukocyte palmitoylated proteins using Li-COR infrared imaging (Akimzhanov and Boehning, 2015). However, alkynyl-palmitate analogs can only incorporate into accessible and reduced cysteine residues of a palmitoylated protein. Stalely palmitoylated proteins, which do not undergo dynamic palmitate turnover, cannot be labeled or purified by this method as their cysteines are not available to conjugate with alkynyl-palmitate analogs.

### Method to Identify Palmitoylation Sites

The palmitoylation sites of the modified protein can be predicted by CSS-Palm 2.0 using a clustering and scoring strategy algorithm, or by other tools like GPS-lipid. To verify the predicted cysteine residues, mutagenesis of cysteine residue(s) is performed, often followed by studying the subsequent impacts on protein palmitoylation level, subcellular localization, stability, and activity. Cysteine residue of a palmitoylated protein is often substituted with alanine, though cysteine-to-serine mutagenesis is also employed by some research groups.

## Protein Palmitoylation Regulation of Leukocyte Function

**FIGURE 2:**
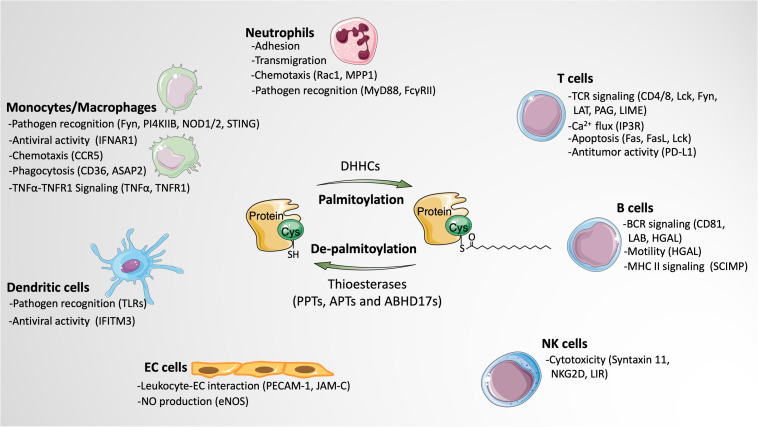
The impact of protein palmitoylation on leukocyte signaling and function. Protein palmitoylation modulates various functions of leukocytes by altering the activities of palmitoylated proteins in different subtypes of leukocytes. Besides, palmitoylation also indirectly affects leukocyte behaviors through regulating the function of endothelial cells. Palmitoylated proteins involved in the particular function are listed in parentheses. Images of cells and proteins were obtained from Smart Servier Medical Art (https://smart.servier.com).

A growing number of palmitoylated proteins have been discovered in leukocytes, many of which play critical roles in regulating various leukocyte functions, including adhesion, transmigration, chemotaxis, phagocytosis, pathogen recognition, T cell receptor (TCR) and BCR signaling activation, cytotoxicity, cytokine production, and apoptosis. Below, we discuss the functional impact of palmitoylation in different types of leukocytes (Figure 2).

### Neutrophils

Neutrophils are the most abundant type of leukocytes in human circulation that act as ongoing surveillance against potential invading pathogens and sterile inflammation. They are among the first responders to sites of infection and contribute to the initiation and propagation of inflammatory cascades (Ma et al., 2019). The neutrophil recruitment cascade is initiated with selectin-mediated slow rolling, followed by β2-integrin-mediated firm adhesion and PECAM-1-mediated transmigration across the endothelium (O’Brien et al., 2003; Mocsai et al., 2015).

#### Neutrophil Adhesion and Transendothelial Migration

Adhesion molecules, such as selectins and integrins, work cooperatively to regulate neutrophil recruitment. Although little is known about palmitoylation of adhesion molecules expressed on the neutrophil surface, it has been seen in many cells and cell fragments that directly interact with neutrophils. For instance, in ECs, PECAM-1 is palmitoylated by DHHC21, and knockdown of DHHC21 greatly reduces the protein level of PECAM-1 and decreases its surface expression (Marin et al., 2012). Another study reports that palmitoylation of PECAM-1 at Cys595 is required for its lipid raft localization (Sardjono et al., 2006). Additionally, P-selectin in platelets mediates the formation of the platelet-neutrophil complex via binding to its ligand PSGL-1 on neutrophils, a process that aids the firm adhesion of neutrophils to activated ECs and thereby promotes leukocyte transmigration (Lisman, 2018). Recent studies have shown that P-selectin undergoes palmitoylation in platelets and that de-palmitoylation of P-selectin by APT1 greatly inhibits the expression of P-selection in platelets (Sim et al., 2007). It is plausible that this palmitoylation-regulated P-selectin change in platelets may affect neutrophil recruitment.

Neutrophil adhesion molecules require partner protein tetraspanins for their stable membrane localization and proper function (Yeung et al., 2018). Tetraspanins are transmembrane proteins that contain a large extracellular loop to interact with adhesion molecules and other tetraspanins, forming a signal transducing complex called tetraspanin-enriched microdomains (TEMs) in cell membrane. Tetraspanins facilitate the recruitment of adhesion molecules to TEMs and amplify their intracellular signals, therefore playing important roles in regulating neutrophil function. For example, CD37^–/–^ neutrophils exhibit an impaired ability to migrate toward chemotactic stimuli and to adhere to postcapillary venules (Wee et al., 2015); CD63 binds to Mac-1 and enhances Mac-1-mediated adhesion function (Skubitz et al., 1996). So far, a large body of tetraspanins, including CD9, CD37, CD53, CD63, CD81, CD82 and CD151, has been reported as palmitoylation substrates (Charrin et al., 2002; Yang et al., 2002; Termini et al., 2014). Investigating whether and how palmitoylation affects tetraspanin function in neutrophils is of great significance for the comprehensive understanding of the regulation of neutrophil functions.

#### Neutrophil Chemotaxis

Once migrated out of microvessels, neutrophils in tissues are capable of moving along chemoattractant gradients toward sites of infection or injury. Neutrophil chemotaxis starts with polarization, characterized by the formation of a leading-edge containing pseudopod and a highly contractile trailing edge (Hind et al., 2016). The migration of the neutrophil depends on dynamic polymerization and depolymerization of actin filament, which pushes the leading-edge forward and retracts the tail at the rear of the cell (Szczur et al., 2006; Mocsai et al., 2015). The key regulators of these coordinated processes are members of the small Rho GTPase family, including Rac, Cdc42, and Rho (Sun et al., 2004; Mocsai et al., 2015).

Rac is essential for neutrophil motility as well as directional sensing during migration. Similar to other members of small GTPase, Rac exists in two states: GTP-bound activated state or GDP-bound de-activated state. During neutrophil migration, GTP-bound Rac1 promotes the activation of p21-activated kinase (PAK) and the reorganization of actin, which subsequently aid membrane protrusion via lamellipodia formation (Filippi et al., 2007). Rac-1 is palmitoylated at Cys178, and the attachment of palmitate requires prior prenylation at Cys189 and a polybasic domain in C-terminal region. Palmitoylation-defective Rac1 (mutation at Cys178) shows reduced ability to load GTP and decreased lipid raft partitioning, which are accompanied by decreased PAK activity and F-actin recruitment at plasma membrane. Cells expressing Cys178 mutant Rac1 display defective spreading and migrate with less directionality, suggesting the essential roles of palmitoylation in regulating Rac1 function and neutrophil migration (Navarro-Lerida et al., 2012). In contrast to Rac, other members of the small GTPase family do not seem to be regulated by palmitoylation. For example, RhoA is a non-palmitoylated small GTPase (Navarro-Lerida et al., 2012), and Cdc42 only undergoes prenylation, with an exception of a brain-specific variant of Cdc42 which is palmitoylated by DHHC8 (Moutin et al., 2017).

In addition to small GTPases, other palmitoylated proteins are also involved in regulating neutrophil chemotactic migration. For example, membrane palmitoylated protein 1 (MPP1 or P55) is essential for the formation of a single pseudopod at the leading edge by confining small GTPase activation and actin polymerization to a localized region. MPP1 knockout neutrophils display multiple transient pseudopods in all directions upon stimulation and fail to perform chemotaxis efficiently (Quinn et al., 2009). However, the impacts of palmitoylation on MPP1 function remain to be addressed in neutrophils.

#### Pathogen Recognition

Neutrophils express several kinds of surface receptors for the recognition of invading pathogens such as Toll-like receptors (TLRs), Fcγ-receptors, and formyl-peptide receptors (Futosi et al., 2013). Growing evidence has shown that many of these receptors and their adaptor proteins are regulated by palmitoylation.

##### Toll-like receptors/MyD88 signaling

Toll-like receptors are responsible for recognizing pathogen-associated molecular patterns (PAMPs) and damage-associated molecular patterns (DAMPs). TLR signaling has previously been shown to be activated by saturated fatty acids even though fatty acids do not bind to TLR, suggesting a ligand-independent TLR activation meditated by fatty acids (Fessler et al., 2009; Lancaster et al., 2018). Further study reveals that MyD88, an adaptor molecule of TLR signaling, is palmitoylated by DHHC6 at Cys113 (Kim et al., 2019). The palmitoylation of MyD88 is required for its binding to IRAK4 and activation of downstream NF-κB. Reducing intracellular palmitate concentration by the inhibition of *de novo* fatty acid synthesis blocks LPS-induced neutrophil activation and MyD88-dependent cytokine production. The critical roles of palmitoylation in TLR/MyD88 signaling is further supported by the fact that the DHHC inhibitor 2-bromopalmitate (2-BP) inhibits NF-κB activation, whereas the APT inhibitor palmostatin B enhances the activation of NF-κB (Kim et al., 2019). In fact, many isotypes of TLRs (TLR 2,5,6,10) are shown to be palmitoylated in other types of leukocytes, which will be discussed in detail later.

##### Fcγ receptors

Neutrophils constitutively express FcγRII (CD32) and FcγRIII (CD16) while transiently expressing FcγRI (CD64) upon activation. Neutrophil Fcγ receptors enable neutrophils to recognize antigens via immunoglobulins (Igs)-mediated interactions and regulate many neutrophil functions (Futosi et al., 2013; Kara et al., 2020). Thus, Fcγ receptors serve as a crucial link between innate immune effector cells and adaptive immune responses. The function of FcγR and its ability to bind to IgG complex requires FcγR partitioning into lipid rafts (Bournazos et al., 2009). It has been shown that the lipid raft targeting of FcγRII is regulated by palmitoylation at Cys208 in its juxtamembrane region; mutation of Cys208 results in diminished translocation of FcγRII to lipid rafts and impaired FcγRII downstream signaling (Barnes et al., 2006).

The palmitoylation of surface receptors like TNFRs, Fas, and CCRs, although not yet tested in neutrophils, have been established in other immune cells (Blanpain et al., 2001; Rossin et al., 2015; Zingler et al., 2019).

### Monocytes/Macrophages

Monocytes and macrophages are critical components of innate immune systems. Monocytes patrol around the circulation and eliminate pathogens via phagocytosis. Once recruited to the sites of inflammation, monocytes differentiate into macrophages or dendritic cells. In addition to phagocytosis, monocytes and macrophages also produce inflammatory mediators and function as antigen presenting cells (APCs). An ABE-coupled proteomic study reveals 98 palmitoylated proteins in macrophages, 48% of which have been reported to be localized in lipid rafts (Merrick et al., 2011). Many of the previously reported palmitoylated proteins participate in various monocyte/macrophage functions, such as syntaxin-7 in phagocytosis, SNAP-23 in TNFα secretion and phospholipid scramblase 3 (PLSCR3) in apoptosis (Vogel and Roche, 1999; He and Linder, 2009; Merrick et al., 2011), suggesting critical roles of palmitoylation in modulating monocyte/macrophage functions.

#### Pathogen Recognition

##### TLR signaling pathway

Emerging evidence demonstrates that palmitoylation is involved in LPS-induced macrophage activation. A proteomics study using RAW264 macrophage-like cells reveals that LPS stimulates global changes in protein palmitoylation, affecting nearly 340 palmitoylated proteins (Kwiatkowska and Ciesielska, 2018). More importantly, TLR signaling in macrophages is regulated by many palmitoylated proteins. Src family kinase Fyn, an inhibitory regulator of TLR4, is palmitoylated at Cys3, a modification required for its lipid raft localization and catalytic function. LPS stimulation increases the level of palmitoylated Fyn in lipid rafts where Fyn mediates the suppression of both NF-κB and IRF3-dependent cytokine production (Borzecka-Solarz et al., 2017). Another LPS-upregulated palmitoylated protein is type II phosphatidylinositol 4-kinase (PI4KIIB). Palmitoylation of PI4KIIB triggers the activation of PI4KIIB and promotes the subsequent synthesis of PI(4,5)P2, a phospholipid compound that can activate TLR4 signaling on its own or through its hydrolytic products DAG, IP3, and PIP3 (Sobocinska et al., 2018).

##### Nucleotide oligomerization domain 1 (NOD1) and NOD2

Nucleotide oligomerization domain1/2 are cytosolic pattern recognition receptors that recognize bacterial peptidoglycans and promote subsequent immune responses such as NF-κB activation and cytokine production. The interaction of NOD1/2 with membrane structures (e.g., plasma membrane and bacterial-containing phagosomes) is essential for their function. NOD1/2 are palmitoylated at multiple cysteine residues: Cys558, 567, and 952 of NOD1 and Cys395 and 1033 of NOD2 (Lu et al., 2019). Administration of 2-BP greatly inhibits the recruitment of NOD1/2 to membrane structures and the production of cytokines in macrophages treated with bacterial peptidoglycans. DHHC5 is responsible for the palmitoylation of NOD1/2; silencing or knockout of DHHC5 abrogates NOD1/2-dependent NF-κB activation in bacterial peptidoglycans-stimulated macrophages (Lu et al., 2019).

##### Stimulator of interferon genes *(STING)*

STING recognizes cyclic dinucleotides of bacteria and DNA viruses, leading to potent cytokine production; it can also be activated by self-DNA that leaks from nucleus or mitochondria, which results in autoinflammatory diseases (Hansen et al., 2019). DHHC3, 7, and 15 mediated-palmitoylation of STING at Cys88 and 91 is required for STING activity and the subsequent promotion of type I interferon (IFN) responses (Mukai et al., 2016). Nitro-fatty acids, which directly target cysteine residues and therefore block STING palmitoylation, greatly reduce the production of type I IFN and IFN-induced CXCL10 and IL-6 in monocytes and macrophages upon virus infection (Hansen et al., 2018).

#### Antiviral Activity

Interferon-α/β receptor (IFNAR) is the receptor for antiviral cytokine type I IFNs that functions as a critical player in combating virus infection (Gonzalez-Navajas et al., 2012). It also possesses anti-bacterial and immunomodulatory properties by inducing the transcription of IFN-stimulated genes (Ivashkiv and Donlin, 2014). Upon binding to type I IFNs, IFNAR undergoes endocytosis and activates receptor-associated Jak kinases, which results in the phosphorylation of signal transducer and activator of transcription 1 (STAT1) and STAT2 and ultimately the transcription of the IFN-stimulated genes (Lee and Ashkar, 2018). Palmitoylation is required for the activation of IFNAR-Jak-Stat pathway. Palmitoylation inhibitor greatly diminishes the endocytosis of IFNAR1 and the phosphorylation of STAT1. Further study reveals that IFNAR1 is palmitoylated at Cys463, and mutation of Cys463 blocks the phosphorylation of STAT1 and 2 and their nuclear translocation, without affecting IFNAR1 endocytosis and stability (Claudinon et al., 2009).

#### Chemotaxis

C-C chemokine receptor type 5 (CCR5) is a chemokine receptor that belongs to the GPCR superfamily. CCR5 regulates macrophage chemotaxis and also functions as a coreceptor for the macrophage-tropic human immunodeficiency (M-tropic HIV) virus entry into monocytes/macrophages and CD4^+^ T lymphocytes (Tuttle et al., 1998). CCR5 is palmitoylated at Cys321, 323, and 324 located between the cytoplasmic end of the seventh transmembrane domain and the C-terminal tail (Percherancier et al., 2001). Mutation of these cysteine residues leads to the greatly reduced surface expression of CCR5. Moreover, palmitoylation prevents CCR5 from degradation, thus stabilizing CCR5; palmitoylation-defective CCR5 displays a 3-fold decease in its half-life compared to WT CCR5. Functional analysis shows that palmitoylation does not affect the binding ability of CCR5 with its agonist macrophage inflammatory protein-1β (MIP-1β). The reduced function of palmitoylation-defective CCR5 is attributed to its impaired surface expression (Blanpain et al., 2001; Percherancier et al., 2001).

#### Phagocytosis

##### CD36

CD36 is the primary scavenger receptor expressed on monocytes and macrophages that facilitates the phagocytosis of pathogens, dead cells, modified low-density lipoproteins, and long-chain fatty acids (Silverstein and Febbraio, 2009). Palmitoylation occurs at both N-terminal (Cys3 and Cys7) and C-terminal (Cys464 and Cys466) residues of CD36 (Meiler et al., 2013). Palmitoylation of CD36 requires ER protein Selk, the cofactor of DHHC6. *Selk*^–/–^ macrophages exhibit reduced membrane expression of CD36 due to the decreased level of CD36 palmitoylation (Meiler et al., 2013). It is possible that Selk tethers DHHC6 to ER membrane via its SH3-binding domain, thus promoting DHHC6-mediated palmitoylation of CD36 in ER of macrophages. However, another study of adipocyte CD36 reveals that DHHC4 and DHHC5 are actually responsible for the palmitoylation of CD36 and its stable membrane localization (Wang J. et al., 2019). In adipocytes, DHHC4-mediated palmitoylation in Golgi targets CD36 to plasma membrane, while DHHC5-mediated palmitoylation in plasma membrane prevents CD36 disassociation with plasma membrane. Whether this modification mechanism also applies to macrophages needs to be further tested.

##### FcγR signaling

Fcγ receptors are also expressed on macrophages, mediating the phagocytosis of IgG-coated pathogens, the secretion of inflammatory mediators, and IgG-dependent elimination of infected cells (Fitzer-Attas et al., 2000). As mentioned in section “Neutrophils- Pathogen Recognition,” palmitoylation is required for FcγR lipid raft partitioning and its binding ability with IgG complex (Barnes et al., 2006). In addition to the receptor itself being palmitoylated, effector protein downstream of FcγR is also palmitoylated in macrophages. Arf-GAP with SH3 domain, ANK repeat, and PH domain-containing protein 2 (ASAP2), is a scaffolding protein that regulates cytoskeletal rearrangement during FcγR-mediated phagocytotic cup formation (Norton et al., 2017). Once phagocytotic cups mature, ASAP2 is cleaved from the cups by calpain-2 to allow efficient phagocytosis to occur. ASAP2 is palmitoylated at Cys86 by DHHC6/Selk in ER, and palmitoylation is required for the cleavage of ASAP2 and the subsequent maturation of the phagocytic cups. Inhibition of ASAP2 palmitoylation by mutagenesis, palmitoylation inhibitor, or Selk mutation leads to prolonged retention of ASAP2 in phagocytic cups and impaired FcγR-mediated phagocytosis (Norton et al., 2017).

#### TNFα-TNFR1 Signaling

TNFα is a multifunctional pro-inflammatory cytokine primarily secreted by macrophages. Newly synthesized TNFα is a transmembrane protein (tmTNFα). Upon stimulation, the ectodomain of tmTNFα is cleaved by TNFα-converting enzyme ADAM17, producing biologically active soluble TNFα (sTNFα). The membrane-bound TNFα fragment is further cleaved by signal peptide peptidase-like 2b (SPPL2b) to generate the intracellular domain of TNFα (ICD-TNFα), which then activates IL-1β promoter. Palmitoylation of TNFα has been reported; the [^3^H] labeled palmitate is detected in tmTNFα but not in sTNFα (Utsumi et al., 2001). Mutation of the palmitoylation site of tmTNFα does not affect ADAM17-dependent cleavage of its ectodomain. However, palmitoylation of tmTNFα regulates the sensitivity of TNFα receptor 1 (TNFR1). Palmitoylation facilitates tmTNFα partitioning to the lipid rafts, where it suppresses the activity of TNFR1 by competing with sTNFα. Whereas, palmitoylation-defective tmTNFα shows reduced ability to block the binding of sTNFα to TNFR1 and the transcription of TNFα target genes (Poggi et al., 2013). Palmitoylation is also required for the enzymatic cleavage of the membrane-bound TNFα fragment by SPPL2b. Mutagenesis of the palmitoylation site of TNFα results in a rapid degradation of the membrane-bound TNFα fragment prior to SPPL2b-mediated cleavage, thus reducing ICD-TNFα production and downstream IL-1β promoter activation (Poggi et al., 2013).

The majority of TNFα biological function is mediated by TNFR1, which belongs to a subgroup of death receptors. Upon binding to TNFα, TNFR1 induces two distinctive downstream signaling pathways depending on its subcellular localization. TNFR1 in plasma membrane signals for NF-κB activation, while internalized TNFR1 activates apoptosis signaling. TNFR1 is palmitoylated at Cys248, which is required for its plasma membrane localization in human monocytic U937 cells (Zingler et al., 2019). Mutagenesis of Cys248 results in decreased TNFR1 expression in plasma membrane, reducing both NF-κB and apoptosis signaling. Furthermore, activated TNFR1 undergoes APT2-mediated de-palmitoylation, which promotes TNFR1 translocation to designated signaling platforms to activate NF-κB. APT2 inhibitor blocks TNFR1 de-palmitoylation and its downstream NF-κB activation but enhances TNFR1-mediated apoptosis signaling (Zingler et al., 2019).

### Dendritic Cells

Dendritic cells (DCs), functioning as the most potent APCs, are specialized to recognize, phagocytize, process, and present antigens to both memory and naïve T cells (Steinman and Banchereau, 2007). There is limited information regarding the roles of palmitoylation in DCs. Two Alk-C16 metabolic labeling-based palmitoyl proteomic studies reveal hundreds of palmitoylated proteins with diverse cellular function in DCs, such as TLR2 for pathogen recognition; IFITM3 for antiviral activity; G protein subunits, N-Ras and Lyn for signal transduction; CD81 for DC migration; and CD80 and 86 for immunomodulation (Yount et al., 2010; Quast et al., 2011; Chesarino et al., 2014; Yeung et al., 2018). Among these proteins, only TLR2 and IFITM3 palmitoylation have been investigated for their functional impact in DCs.

#### Pathogen Recognition

##### Toll-like receptors

In DCs, TLR2 is found to be palmitoylated on Cys609 at the cytoplasmic edge of the transmembrane domain (Chesarino et al., 2014). Multiple DHHCs (DHHC 2, 3, 6, 7, 15) are responsible for the palmitoylation of TLR2. Inhibition of TLR2 palmitoylation either by 2-BP or mutation of the palmitoylation site leads to the decreased surface expression of TLR2 in DCs, without affecting the overall protein level and stability of TLR2. Blocking TLR2 palmitoylation also inhibits microbial ligands-induced activation of NF-κB and production of IL-6 and TNFα, which is, at least partially, due to the insufficient surface expression of TLR2. Similar to TLR2, other TLRs (TLR5,6,10) have been shown to be palmitoylated in DCs (Chesarino et al., 2014). Whether and how palmitoylation regulates their function remain to be addressed.

#### Antiviral Activity

Interferon-induced transmembrane protein 3 (IFITM3), an INF-stimulated effector protein, plays important roles in restricting virus infection in host cells. Upon virus infection, IFITM3 interacts with the incoming virus particles and directs them to lysosomes for elimination. The essential roles of palmitoylation in the antiviral activity of IFITM3 have been well-recognized (Yount et al., 2010; Spence et al., 2019). In DCs, IFITM3 is palmitoylated on Cys71, Cys72 and Cys105 in the transmembrane proximal region (Yount et al., 2010). Mutation of IFITM3 palmitoylation sites does not affect the expression or subcellular localization of IFITM3 in DCs at steady state; however, palmitoylation-defective IFITM3 exhibits a greatly reduced ability to cluster with virus particles and impaired antivirus activity (Yount et al., 2010; Spence et al., 2019).

### T Cells

T cells coordinate a variety of adaptive immune responses. The pivotal roles of protein palmitoylation in regulating T cell functions have been well recognized.

#### T Cell Receptor Signaling

T cells are activated by the binding of TCRs to the major histocompatibility complex (MHC):antigen peptide complex. Briefly, the TCR-pMHC engagement facilitated by co-receptors CD4/CD8 activates tyrosine kinase Lck and Fyn to phosphorylate the TCR/CD3 ζ-chain, recruiting tyrosine kinase ZAP-70 to phosphorylate transmembrane adaptor proteins (LAT, PAG, *etc.*). This initiates the propagation of several signaling pathways and eventually leads to the activation of the transcription factors NFAT, NF-κB, and AP-1. One study has shown that 2-BP disrupts signaling protein localization and blocks TCR signaling as evidenced by reduced tyrosine phosphorylation and impaired calcium influx (Webb et al., 2000). Moreover, several palmitoyl proteomic studies reveal that although T-cell receptor itself does not undergo palmitoylation, TCR co-receptors CD4, CD8, Src family kinases Lck and Fyn, and adaptor proteins LAT and PAG are all palmitoylated (Martin and Cravatt, 2009; Morrison et al., 2015). How palmitoylation regulates the function of TCR signaling proteins has been previously reviewed (Bijlmakers, 2009; Ladygina et al., 2011); therefore, it will only be briefly discussed in this section. Signaling effectors mentioned in previous sections like Ras and Rac will not be covered again in this section.

##### T cell receptor coreceptors CD4/CD8

The extracellular domain of CD4 and CD8 binds to the non-polymorphic regions of Class II and Class I MHC on APCs, respectively, facilitating the interaction of TCR with pMHC. Meanwhile, their intracellular cytoplasmic tails are closely associated with Lck (Artyomov et al., 2010; Courtney et al., 2018). Palmitoylation of CD4 occurs at Cys394 and Cys397 residues adjacent to the transmembrane domain (Crise and Rose, 1992). Palmitoylation functions as the lipid raft targeting signal of CD4, facilitating its partitioning in lipid raft and clustering with TCR and PKCθ there (Balamuth et al., 2004). CD4 also serves as a key coreceptor for HIV entry in T cells, and current research indicates that CD4 palmitoylation is not required for this function (Bijlmakers, 2009). CD8 is heterodimer consisting of CD8α and CD8β. Species-specific palmitoylation of CD8 has been reported. In mice, only CD8β, but not CD8α, undergoes palmitoylation on Cys179 in its cytoplasmic domain (Arcaro et al., 2001). In contrast, human CD8β contains two palmitoylated cysteine residues, and human CD8α is also palmitoylated. In mouse T cells, mutation of Cys179 abolishes CD8 lipid raft localization and impairs its association with Lck (Arcaro et al., 2001). Unexpectedly, palmitoylation of human CD8 does not seem to play a role in targeting CD8 to lipid raft (Pang et al., 2007). Instead, lipid raft targeting of CD8 solely relies on the positively charged amino acids in CD8β (Bijlmakers, 2009). The impact of palmitoylation on other functions of CD8 still remains unclear.

##### Src family kinases

Lck is actively involved in TCR signaling as well as T cell development (Molina et al., 1992; Palacios and Weiss, 2004). Lck is a dually acylated protein (Resh, 1994). Upon synthesis, Lck is myristoylated at Gly2, which is required for the subsequent palmitoylation at Cys3 and Cys5. Stable association between Lck and plasma membrane, which is critical for the activity of Lck, is mediated by palmitoylation at both Cys3 and Cys5 (Yurchak and Sefton, 1995). Multiple DHHCs (2, 3, 17, 18, 21) are responsible for the palmitoylation of Lck (Aicart-Ramos et al., 2011; Zeidman et al., 2011; Akimzhanov and Boehning, 2015). Unexpectedly, Lck is mainly localized outside of lipid rafts in steady state T cells, yet T cell activation accelerates the palmitate turnover of Lck, which promotes the translocation of Lck into lipid rafts to phosphorylate Fyn and other substrates (Kosugi et al., 2001). Lck with mutation at both palmitoylation sites appears in a soluble form and is unable to interact with CD4 and promote downstream signaling events (Kabouridis et al., 1997). Fyn is another highly expressed Src family kinase in T cells. The absence of Fyn leads to reduced responses to TCR stimulation suggesting that Fyn is also involved in TCR signaling (Rodolfo et al., 1991; Tsygankov et al., 1996; Yasuda et al., 2002). Similar to Lck, Fyn also undergoes both myristoylation and palmitoylation. Attachment of palmitate can occur on both Cys3 and Cys6, although the majority of Fyn is only palmitoylated on Cys3 (Liang et al., 2001). The attached palmitate on Cys3 (with a half-life of 1.5–2 h) is important for membrane association and lipid raft sequestering of Fyn (Wolven et al., 1997). DHHC2,3,7,10,15,20 and 21 have all been shown to catalyze the palmitoylation of Fyn (Mill et al., 2009). In resting T cells, Fyn, unlike Lck, mainly resides in lipid rafts. Upon TCR ligation, Fyn is activated by Lck in lipid rafts which, in turn, catalyzes the phosphorylation of the TCR/CD3 ζ-chain and transduces the signals to downstream effectors (Filipp et al., 2003).

##### Transmembrane adaptor proteins (TRAPs)

TRAPs serve as the functional connection between TCR complex and intracellular signaling molecules. Following TCR ligation, TRAPs are phosphorylated by activated Src family kinases and ZAP-70, allowing the subsequent recruitment of cytoplasmic signaling effectors and the propagation of TCR-elicited signals into cytoplasm (Horejsi et al., 2004). Among all TRAPs, LAT is indispensable for peripheral T cell activation due to its ability to recruit and activate many key signaling molecules including PLCγ1, PI3K, Grb2, Grads, and SLP76 (Horejsi, 2004). LAT is palmitoylated at Cys26 and Cys29 in the juxtamembrane Cys-X-X-Cys motif. LAT palmitoylation is essential for the stability of LAT and its association with plasma membranes; mutation in two palmitoylation sites results in a remarkably reduced LAT expression and the retention of LAT in Golgi complex (Shogomori et al., 2005; Tanimura et al., 2006). Moreover, palmitoylation-defective LAT is not capable of interacting with TCR complex or recruiting PLCγ and Grb2 to propagate signals (Harder and Kuhn, 2000). T cells expressing palmitoylation-defective LAT display impaired Ca^2+^ influx, ERK activation, and transcription factor induction upon T cell activation (Zhang et al., 1999). Furthermore, LAT palmitoylation is closely related to T cell anergy. Compared to control T cells, anergic T cells display profoundly reduced LAT palmitoylation, which leads to reduced LAT recruitment to immunological synapses and lipid rafts (Hundt et al., 2006). Functionally, LAT in anergic T cells shows a reduced phosphorylation level upon TCR stimulation and an impaired ability to activate downstream PLCγ and PI3K (Hundt et al., 2006; Ladygina et al., 2011).

Other TRAPs like phosphoprotein associated with GEMs (PAG) and Lck-interacting membrane protein (LIME) are also palmitoylated at the same Cys-X-X-Cys motif in T cells, and both are concentrated in lipid rafts (Ladygina et al., 2011). PAG, also known as Csk-binding protein (Cbp), acts as negative regulator of T-cell signaling. PAG is capable of recruiting Csk to inhibit Src family kinases via phosphorylating tyrosine residues in their C-terminal regulatory domains. Upon TCR ligation, PAG rapidly releases Csk, resulting in the activation of Lck and Fyn (Davidson et al., 2003). PAG with a mutation in its palmitoylation motif displays normal expression level and plasma membrane localization, but it no longer resides in lipid rafts. Although it is able to recruit Csk, palmitoylation-defective PAG cannot inhibit proximal TCR signaling (Posevitz-Fejfar et al., 2008). Similar to PAG, LIME can also bind to Csk and Lck, yet the biological function of LIME in T cells remains unclear. One study has shown that LIME contributes to the activation of ERK and JNK and the production of IL-2 in Jurkat T cells (Hur et al., 2003). The attached palmitates at Cys28 and Cys31 in the Cys-X-X-Cys motif act as lipid raft targeting signals for LIME (Hur et al., 2003). However, the functional significance of palmitoylation on LIME has not yet been investigated.

#### Calcium Flux

Upon TCR activation, intracellular signaling effector PLCγ hydrolyzes PI(4,5)P2 to produce DAG and IP3; IP3, in turn, triggers Ca^2+^ release from ER stores via binding to IP3Rs on ER membrane (Nagaleekar et al., 2008). IP3Rs are palmitoylated by DHHC6/Selk complex in ER. The putative palmitoylation sites are reported to be at Cys56, 849, and 2214, which needs further confirmation (Fredericks et al., 2014). Knockdown of DHHC6 significantly reduces the expression of IP3R on ER membrane and attenuates IP3R-dependent Ca^2+^ flux in Jurkat T cells (Fredericks et al., 2014). The proper function of DHHC6 requires binding to its cofactor Selk via the SH3-binding domain in Selk. Selk deficiency also leads to decreased IP3R level, probably due to increased IP3R proteolysis. In fact, this DHHC/Selk-dependent regulation of Ca^2+^ release is shared by many immune cells. In Selk knockout mice, receptor-mediated Ca^2+^ flux is impaired in T cells, neutrophils, and macrophages, resulting in disrupted T cell proliferation, neutrophil migration, and macrophage oxidative burst (Verma et al., 2011).

#### Apoptosis

The ligation of Fas and FasL induces apoptosis of T cells that recognize self-antigens or are activated repeatedly, key for eliminating autoreactive T cells and preventing autoimmune diseases (Volpe et al., 2016). Palmitoylation plays critical roles in regulating the Fas-FasL signaling pathway in T cells as both Fas and FasL are palmitoylation substrates. Fas (also known as CD95, or APO1 or TNFRSF6) is a type I transmembrane protein that contains a death domain in its cytoplasmic region. Upon binding to FasL, Fas recruits death-inducing signaling complex (DISC), which, in turn, activates caspase-3 and induces programmed cell death. Promoting apoptosis signals requires Fas partition into actin cytoskeleton-linked lipid rafts and then internalization, both of which are regulated by Fas palmitoylation (Rossin et al., 2015). Mouse Fas is palmitoylated at Cys194 adjacent to the transmembrane domain, while human Fas is palmitoylated at Cys199. One study shows that DHHC7 is responsible for the palmitoylation of Fas, and this modification stabilizes Fas by preventing its degradation by lysosomes (Rossin et al., 2015). Palmitoylation-defective Fas exerts severely reduced lipid raft localization, which impairs DISC assembly, Fas internalization, and T cell apoptosis (Chakrabandhu et al., 2007; Cruz et al., 2016). The impaired apoptosis-inducing ability of palmitoylation-defective Fas can also be observed in other Fas-expressing immune cells, like B cells and dendritic cells (Cruz et al., 2016).

Palmitoylation also participates in the signaling propagation downstream of Fas ligation, including Lck activation, PLC-γ1 phosphorylation, and proapoptotic Ca^2+^ release. 17-ODYA metabolic labeling reveals that Fas ligation leads to a rapid increase in Lck palmitoylation followed by quick de-palmitoylation, catalyzed by DHHC21 and APT1, respectively (Akimzhanov and Boehning, 2015). This rapid dynamic of Lck palmitoylation matches the activation kinetics of other signaling molecules subsequent to Fas stimulation. Re-expressing palmitoylation-defective Lck is unable to induce Fas-mediated Ca^2+^ release, caspase-3 activation, or cell death in Lck-deficient Jurkat T cells (Akimzhanov and Boehning, 2015). It is worth noting that, in abnormally or excessively activated T cells, the membrane FasL can be cleaved by metalloproteases (e.g., ADAM10 and AMDA17) to generate sFasL. sFasL binding to Fas is unable to activate Fas, thus sFasL serves as a competitor of FasL to suppress FasL-induced apoptosis (Suda et al., 1997). Shedding of FasL requires the colocalization of FasL, metalloproteinases, and Lck in lipid rafts. The lipid raft targeting of both FasL and Lck depends on palmitoylation, with FasL being palmitoylated on Cys82 and Lck at Cys3 and Cys5. Inhibition of palmitoylation with 2-BP remarkably blocks FasL shedding in T cells (Ebsen et al., 2015).

#### Antitumor Activity

Programmed cell death 1 (PD1), an inhibitory surface receptor of T cells, suppresses T cell activation through binding to its ligands programmed-death ligand 1 (PD-L1) and PD-L2, playing essential roles in self-tolerance. Many tumor cells often express high level of PD-L1, which inhibits the antitumor responses of T cells such as cytokine production and cytotoxicity (Juneja et al., 2017). PD-L1 is palmitoylated by DHHC3 in its cytoplasmic domain, and the attachment of palmitic acid stabilizes PD-L1 due to decreased ubiquitination. Inhibiting PD-L1 palmitoylation via 2-BP or silencing of DHHC3 greatly reduces the expression of PD-L1 in tumor cells and therefore promotes T cell-mediated antitumor immunity (Yao et al., 2019).

### B Cells

B cells are specialized in producing highly specific antibodies in response to diverse foreign antigens, thus playing critical roles in adaptive immunity. They can also serve as APCs and produce a variety of cytokines (IL-6, IL-10, CXCL13, and CCL19, etc.), influencing the function of other immune cells (Fillatreau, 2018). Palmitoylation has been studied less in B cells compared to T cells. An ABE-based palmitoyl proteomic study in human B cells reveals over 100 palmitoylated proteins or candidates that are known to participate in diverse cellular events of B cells (e.g., signal transduction, vesicle fusion, molecular transport, metabolism and immune responses), highlighting the functional significance of palmitoylation in regulating B cell behaviors (Ivaldi et al., 2012).

#### B Cell Receptor Signaling

##### B cell receptor (BCR) coreceptors

B cell receptors binding to complement-tagged antigens requires the BCR coreceptor complex CD19/CD21/CD81 as they significantly lower the threshold of BCR activation to antigens. Upon BCR ligation, the BCR-CD19/CD21/CD81 complex translocates to lipid rafts for the efficient propagation of BCR-elicited signals. Similar to TCR, none of the BCR subunits are palmitoylated; yet, its co-receptor CD81 is a well-established palmitoylation substrate. CD81, which belongs to the tetraspanin superfamily, is palmitoylated at six cysteine residues in its juxtamembrane domain (Delandre et al., 2009; Yeung et al., 2018). The association of BCR and CD19/CD21/CD81 following BCR ligation rapidly promotes the palmitoylation of CD81, which regulates BCR signaling at many levels (Flaumenhaft and Sim, 2005). First, the prolonged residency of BCR-CD19/CD21/CD81 in lipid rafts depends on the palmitates attached to CD81 (Cherukuri et al., 2004). Moreover, the binding of CD81 to other co-receptors requires palmitoylation. Palmitoylation-defective CD81 exhibits impaired association with CD19 (Delandre et al., 2009). Finally, palmitoylation of CD81 affects the ability of CD81 to recruit the downstream signaling molecule 14-3-3, an adaptor protein regulating the activities of PKCs and PI3K (van Hemert et al., 2001). Inhibition of palmitoylation by 2-BP blocks BCR signaling activation, as evidenced by reduced PLCγ2 phosphorylation and Ca^2+^ mobilization (Cherukuri et al., 2004). Interestingly, CD81 palmitoylation is significantly decreased in the presence of the oxidative stress, indicating CD81 may be responsible for redox stress-induced B cell responses (Clark et al., 2004).

##### Adaptor proteins

Linker for activation of B cells (LAB), also known as non-T cell activation linker (NTAL), is a member of TRAPs that regulates BCR signaling. Similar to LAT, LAB is palmitoylated in a cytoplasmic juxtamembrane Cys-X-X-Cys motif and is therefore enriched in lipid rafts (Lupica et al., 1990; Brdicka et al., 2002). However, the functional impacts of LAB palmitoylation have not been explored.

#### Motility

Human germinal center-associated lymphoma (HGAL) is a newly identified adaptor protein regulating BCR signaling and B cell motility. HGAL is located in both plasma membrane and cytoplasm. Plasma membrane HGAL enhances BCR signaling by increasing Syk activity and its downstream Ca^2+^ mobilization (Romero-Camarero et al., 2013); cytosolic HGAL inhibits spontaneous and chemoattractant-induced B cell motility via suppressing the interaction between myosin and actin. The shuttling of HGAL between the cytoplasm to the plasma membrane requires the acylation modification as HGAL does not contain transmembrane domains. [^3^H] palmitate labeling reveals that HGAL is palmitoylated at the Cys43-F-Cys45 motif (Lu et al., 2015). Upon BCR ligation, palmitoylation, together with myristoylation at Gly2, facilitate the translocation of HGAL into lipid rafts where it binds to Syk and enhances Syk activity. HGAL with mutation at both palmitoylation sites fails to promote the phosphorylation of Syk and downstream Ca^2+^ influx in the presence of BCR stimulation. However, acylation site mutation of HGAL results in the accumulation of HGAL in cytoplasm and therefore induces a further reduction in B cell motility in response to chemoattractants (Lu et al., 2015).

#### MHC II Signaling

SLP65/SLP76, Csk-interacting membrane protein (SCIMP) belongs to the TRAP family and is involved in MHC II signaling in B cells during antigen presentation. SCIMP is mainly localized in lipid rafts and directly binds to Lyn. Upon MHC II stimulation, SCIMP is phosphorylated by Lyn, which, in turn, serves as a recruiting platform to interact with SLP65/SLP76, Csk, CD81, and Grb2 and regulates downstream MHC II signals. SCIMP is palmitoylated at its juxtamembrane palmitoylation motif (Cys-X-Cys; Draber et al., 2011. The roles of palmitoylation in SCIMP function, however, remain unclear.

Other palmitoylated proteins have been identified and confirmed in B cells (Ivaldi et al., 2012). For example, CD20, a four-transmembrane protein that participates in B cell development and differentiation, is palmitoylated on Cys111 and Cys220 in its intracellular domain; the low-affinity IgE receptor FcεRII (CD23) is a transmembrane glycoprotein involved in the regulation of IgE synthesis. Palmitoylation can occur at both Cys17 and Cys18 within the intracellular domain of CD23 (Ivaldi et al., 2012). The functional impacts of palmitoylation on these proteins have not yet been investigated. Since B cells share several common signaling pathways with other immune cells, many of the aforementioned palmitoylated proteins like Ras, Gα, Lck and Fyn, IP3R, and surface receptors will not be discussed again in this section.

### Natural Killer Cells

Natural killer cells are potent cytotoxic lymphocytes that mediate the elimination of potentially harmful cells such as pathogen-infected cells, tumor cells, and cells from transplanted organs. The cytotoxicity is tightly controlled by activating receptors (e.g., NKG2D) that recognize cellular stress ligands and by inhibitory receptors (e.g., LIR and KIR) that recognize MHC class I proteins on target cells (Pegram et al., 2011). So far, there are only a few studies demonstrating that palmitoylation regulates NK cytotoxicity, by either modulating secretory lysosome exocytosis in NK cells or affecting NK receptor ligands in target cells.

#### Syntaxin 11

Upon ligation of activating receptors, NK cells, together with its target cells, form immunological synapses at contact points. NK cells undergo secretory lysosome exocytosis, during which the secretory lysosomes move toward immunological synapses and fuse with plasma membrane to release their cytotoxic contents (e.g., perforin and granzymes). Syntaxin 11, a soluble N-ethylmaleimide-sensitive factor attachment protein receptor (SNARE), catalyzes the membrane fusion reaction via its interaction with trans-SNARE in the opposing membrane. Syntaxin 11 is palmitoylated in its C-terminal cysteine-enriched domain in NK cells (Hellewell et al., 2014). The attached palmitate is required for the association of syntaxin 11 with cell membrane as syntaxin 11 does not contain a transmembrane domain. Mutation of cysteine residues in syntaxin 11 leads to the retention of syntaxin 11 in cytoplasm, causing impaired secretory lysosome exocytosis and reduced NK cytotoxicity (Hellewell et al., 2014).

#### Natural killer group 2 member D (NKG2D)

NKG2D is an activating receptor abundantly expressed on NK cells, mediating the cytotoxicity of NK cells (Wensveen et al., 2018). NKG2D itself has not been reported to be palmitoylated; however, its ligand MHC-class-I-related chain A (MICA) undergoes palmitoylation modification at Cys306 and Cys307 in its cytoplasmic tail (Aguera-Gonzalez et al., 2011). The majority of MICA is expressed on cell membranes of stressed target cells, yet MICA can also be clustered into lipid rafts to be cleaved by metalloproteases to produce soluble MICA, a negative regulator of NKG2D expression and activation. Palmitoylation is required for MICA translocation to lipid rafts and its subsequent shedding; mutation of palmitoylation sites greatly reduces the production of soluble MICA (Aguera-Gonzalez et al., 2011).

#### Leukocyte ig-like receptor (LIR)

LIR is an inhibitor receptor on NK cells that binds to MHC class I proteins on target cells, suppressing NK cell cytotoxicity and preventing NK cells from lysing normal “self” cells. MHC class I proteins are glycoproteins expressed on the plasma membrane of all nucleated cells in vertebrates (Hewitt, 2003). Some of the MHC class I proteins share the same unique cysteine residues in their cytoplasmic tails. Mutation of these cysteine residues abolishes the palmitoylation modification on these proteins. Concomitantly, their surface expression, extracellular confirmation, and interaction with LIR are all disrupted by cysteine residue mutation (Gruda et al., 2007). Whether palmitoylation plays a causative role in these impairments needs to be further elucidated.

## Indirect Regulation of Leukocyte Function by EC Protein Palmitoylation

Palmitoylation also regulates the function of non-immune cells that interact with leukocytes, thus indirectly affecting leukocyte functions and behaviors. A key initiation event of inflammation is the engagement of ECs with leukocytes. Our previous observations with ECs indicate that inflammatory mediators trigger a rapid increase in endothelial palmitoylated proteins (Beard et al., 2016). Palmitoylation is involved in many endothelial responses, such as the surface expression of adhesion molecules, secretion of vasoactive molecules (e.g., nitric oxide), and EC junction integrity. In our previous study, we show that the palmitoylation inhibitor 2-BP greatly attenuates the ICAM-1 surface expression of ECs in response to IL-1β and blocks leukocyte-EC adhesion *in vitro*. In rats subjected to LPS stimulation, leukocyte slow-rolling and adhesion to postcapillary venules are remarkably inhibited by 2-BP. DHHC21 serves as one responsible PAT mediating leukocyte adhesion since DHHC21 loss-of-function greatly reduces the number of slow-rolling and adherent leukocytes in LPS-treated mice (Beard et al., 2016). In addition, junctional adhesion molecule c (JAM-C) is another palmitoylation-modified adhesion molecule that regulates leukocyte behaviors. JAM-C belongs to the immunoglobin superfamily and contains the PDZ-binding motif. Endothelial JAM-C is located at cell-cell contact, especially in tight junctions, interacting with other PDZ motif-containing molecules like JAM-B and ZO-1. JAM-C and JAM-B promote leukocyte adhesion and transmigration due to their ability to act as counterreceptors for Mac-1 and VLA-4, respectively (Bradfield et al., 2007; Scheiermann et al., 2009). JAM-C is palmitoylated at Cys264 and 265 by DHHC7, and the palmitoylation of JAM-C is required for its tight junction localization. Functionally, palmitoylation of JAM-C affects the transmigration of lung cancer cells (Aramsangtienchai et al., 2017). However, the functional impacts of JAM-C palmitoylation on leukocyte adhesion and transmigration need to be further investigated.

Endothelial nitric oxide synthase, which produces nitric oxide (NO), is a well-established palmitoylated substrate. Beyond its vasodilation function, eNOS-derived NO also exhibits important anti-inflammatory effects. Many studies have established that NO suppresses leukocyte adhesion, partly due to its ability to decrease the expression of adhesion molecules both on ECs and leukocytes, like VCAM-1, MCP-1 and CD11/CD18 (Shu et al., 2015). eNOS is primarily localized in Golgi and caveolae (Liu et al., 1996). It is dually acylated by both N-myristoylation at Gly2 and palmitoylation at Cys15 and Cys26 (Sessa et al., 1993; Liu et al., 1995). Palmitoylation of eNOS in ECs is highly dynamic with a turnover rate of 45 min. Multiple DHHCs (2,3,7,8,21) participate in the palmitoylation of eNOS. Inhibiting palmitoylation of eNOS via knockdown of DHHC21 leads to eNOS mislocalization and impaired production of NO in ECs (Liu et al., 1995; Fernandez-Hernando et al., 2006). It is plausible that this reduction in NO caused by DHHC21 knockdown then alters leukocyte function and behaviors.

## Concluding Remarks and Perspective

The dynamic reaction of palmitoylation and de-palmitoylation rapidly and effectively regulates protein conformation, localization, lipid raft partitioning, intracellular trafficking, stability, and interactions with other proteins in leukocytes. Protein palmitoylation has great impact on many aspects of leukocyte function, including adhesion, transmigration, chemotaxis, phagocytosis, pathogen recognition, TCR and BCR signaling activation, cytotoxicity, cytokine production, and apoptosis. Despite the advancement in the field of palmitoylation, many critical issues remain. First, hundreds of putative palmitoylated proteins have been identified in leukocytes via large-scale palmitoyl proteomics, yet only a very small portion of them have been validated for functional impact. The large body of unexplored data contains extensive novel knowledge on palmitoylation in leukocytes that requires further investigation. Second, current understanding on how palmitoylation affects leukocyte proteins has been primarily concentrated on palmitoylation-mediated protein partitioning into membrane and/or lipid rafts; yet other regulatory mechanisms of palmitoylation have been studied to a much lesser extent. To acquire more comprehensive knowledge on palmitoylation, it will be essential to also understand its relationship with other PTMs (e.g., phosphorylation and ubiquitination), its impact on protein conformation, and its ability to mediate protein-protein interaction. Finally, our current knowledge on enzymes that catalyze palmitoylation and de-palmitoylation is still very limited, and the molecular basis of substrate recognition remains largely unknown. There is an urgent need for developing DHHC-selective inhibitors, as the most commonly used inhibitor 2-BP has been shown to exert off-target effects by interfering with fatty acid metabolism (Davda et al., 2013). Mechanisms regulating DHHC activity and substrate specificity will serve as a key in the development of DHHC specific inhibitors.

Taken together, palmitoylation influences many aspects of leukocyte function by altering protein localization and activity. Targeting palmitoylation may serve as a promising therapeutic strategy for aberrant immune responses during inflammatory injury, immune disorders, and infectious diseases.

## Author Contributions

XY performed literature search, drafted the manuscript, and prepared the figures. YM, VC, and SYY edited and revised the manuscript. EZ edited the manuscript and revised the figures. SYY initiated, directed, and sponsored the work throughout all levels of development. All authors approved it for publication.

## Conflict of Interest

The authors declare that the research was conducted in the absence of any commercial or financial relationships that could be construed as a potential conflict of interest.
